# The Influence of Knee Position on Ultrasound Imaging of Femoral Cartilage in Individuals with Anterior Cruciate Ligament Reconstruction

**DOI:** 10.1177/19476035231205682

**Published:** 2023-10-16

**Authors:** Harry S. Battersby, Skylar C. Holmes, Eric J. Shumski, Caitlyn E. Heredia, Steven A. Garcia, Derek N. Pamukoff

**Affiliations:** 1 School of Kinesiology, Western University, London, ON, Canada; 2 Department of Kinesiology, University of Massachusetts Amherst, Amherst, MA, USA; 3 Department of Kinesiology, University of Georgia, Athens, GA, USA; 4 Nashville Soccer Club, Nashville, TN, USA; 5 School of Kinesiology, University of Michigan, Ann Arbor, MI, USA

**Keywords:** gait, osteoarthritis, injury, echo intensity

## Abstract

**Background:**

Articular cartilage is important for knee function and can be imaged using ultrasound. The purpose was to compare femoral cartilage thickness and echo intensity (EI) measured at 90° and 140° of knee flexion and between limbs in a cohort with unilateral anterior cruciate ligament reconstruction (ACLR). We also examined associations between gait biomechanics and cartilage outcomes.

**Methods:**

Twenty-seven individuals with primary unilateral ACLR participated (12 men, 15 women; age = 22.3 ± 3.8 years; time since ACLR = 71.2 ± 47.2 months). Ultrasound was used to obtain femoral cartilage measurements. Gait outcomes included peak KFA (knee flexion angle) and peak external knee flexion moment (KFM). Cartilage outcomes were compared using a 2 (*position*) × 2 (*limb*) repeated measures ANOVA (analysis of variance). Gait and cartilage associations were assessed using linear regression.

**Findings:**

There were no position × limb interactions for any cartilage outcome (all *P* > 0.05). Medial (*P* = 0.038) and central cartilage (*P* < 0.001) were thicker, whereas central (*P* = 0.029) and lateral cartilage EI (*P* = 0.003) were lower when measured at 90° than those at 140° of knee flexion. Medial cartilage was thicker in the ACLR than that in the contralateral limb (*P* = 0.016). A larger KFM was associated with thicker medial cartilage (ΔR^2^ = 0.146, *P* = 0.021) and central cartilage (ΔR^2^ = 0.159, *P* = 0.039) measured at 140° of knee flexion in the ACLR limb but not at 90°.

**Interpretation:**

Findings suggest that imaging position influences cartilage thickness and EI measurements in individuals with ACLR and should be considered in study designs and clinical evaluation. A greater KFM was associated with thicker cartilage within specific portions of the distal femur.

## Introduction

Anterior cruciate ligament reconstruction (ACLR) contributes to an increased incidence of both tibiofemoral^1^ and patellofemoral knee osteoarthritis (KOA).^2^ The link between ACLR and tibiofemoral KOA has been well established as the risk increases 3-fold in the affected compared with the contralateral limb.^3^ However, patellofemoral KOA may be more strongly associated with KOA symptoms,^4^ with studies showing that 11 years post ACLR more than 40% of individuals suffer from radiographic KOA.^5^ Radiography is used to evaluate KOA by measuring joint space narrowing.^6^ However, radiography only provides an indirect assessment of cartilage thinning that may lack sensitivity to detect early changes.^7^ For example, femoral cartilage thinning has been observed using MRI within the first 2 years after ACLR,^8^ and thus, cartilage imaging may be useful for clinicians and researchers when assessing long-term joint health after traumatic knee injury.

Ultrasonography is a reliable and valid method for measuring femoral cartilage thickness compared with MRI and cadavers.^9^ Femoral cartilage is commonly viewed transversely over the superior border of the patella with the knee at 140° or at maximum flexion when evaluated using ultrasound to obtain an unimpeded view of the load-bearing region of the femoral trochlea that articulates with the patellofemoral joint.^9,10^ However, this position only views a singular portion of the anterior distal femur. Cartilage morphology is not uniform across the distal femur and thicker cartilage is found in areas that experience higher compressive forces.^11^ Mechanical load and compression during daily activities, such as gait, are critical for cartilage maintenance because they stimulate anabolic activity.^12^

Ultrasound also measures tissue echo intensity (EI), which has been used to measure composition of other soft tissues, such as collagen concentration, water content, and adiposity.^13,14^ Alteration of cartilage composition occurs before cartilage thinning in KOA, with degenerating cartilage having increased water content.^15^ As water is hypo-echoic, a decrease in cartilage EI has been associated with an increased risk of having medial femoral arthroscopic cartilage damage.^16^ Given that an increased cartilage water content can occur before cartilage thinning, and that EI has been associated with compositional features in other soft tissues, assessing cartilage EI after ACLR may provide a complementary assessment to thickness to comprehensively evaluate cartilage health.

Individuals with ACLR have altered gait biomechanics that may contribute to cartilage degeneration in the injured compared with the contralateral limb.^17^ For instance, individuals with ACLR have smaller knee flexion angles (KFAs) and external peak knee flexion moments (KFMs) in the injured compared with the uninjured limb during gait.^18^ Reductions in these sagittal plane gait mechanics are indicative of a quadriceps avoidance gait strategy,^19^ and may contribute to cartilage thinning.^20^ When using ultrasound, a larger KFM and KFA have been associated with thicker medial anterior femoral cartilage in limbs with ACLR.^21,22^ However, associations between sagittal plane knee mechanics and cartilage outcomes were moderate and had substantial portions of variance unexplained that may be due to joint position during image acquisition. When viewing the cartilage near maximum knee flexion (~140º),^23^ the patella may translate inferiorly over the femur, such that the portion of femoral cartilage in view is a central region of the femur. Conversely, it is likely that the central portion of femoral cartilage is not in contact with either the patella^24^ or the tibia^25^ when the knee is flexed between 20° and 60° during the gait cycle,^26^ and thus experiences minimal compressive force in either articulation. However, slight extension from maximum flexion may provide a vantage to view anterior femoral cartilage that experiences larger patellofemoral compressive force during gait.^27^ Previous research has evaluated cartilage on the medial femoral condyle at 90° of knee flexion using a transverse probe placement.^21^ To our knowledge, no study has evaluated the influence of knee position during image acquisition on femoral trochlea cartilage characteristics using a suprapatellar probe placement.

The purpose of this study was to compare femoral cartilage thickness and EI at 140° and 90° of knee flexion, and between limbs in a cohort with unilateral ACLR. A secondary purpose was to examine the associations between peak KFM, KFA, and during gait and cartilage outcomes from both positions. We hypothesized that cartilage would be thicker when measured at 90° than at 140° of knee flexion, and thicker in the uninjured compared with the injured limb. We also hypothesized that EI would be lower at 140° than at 90° of knee flexion and lower in the ACLR limb compared with the contralateral limb. Finally, we hypothesized a larger KFA and KFM would be associated with thicker cartilage and higher cartilage EI from both measurement positions within the ACLR limb.

## Methods

### Participants

Twenty-seven individuals (12 men, 15 women; age = 22.3 ± 3.8 years; body mass index = 25.8 ± 6.0 kg/m^2^; time since ACLR = 71.2 ± 47.2 months; 56% with concomitant meniscal pathology; 10 hamstrings tendon autograft, 9 patella tendon autograft, 8 allograft) with primary unilateral ACLR participated in this study. An *a priori* sample size estimate determined that 24 participants would be needed to achieve 80% power, assuming a moderate difference between measurement positions and limbs (f = 0.25, α = 0.05, β = 0.20, correlation between measures = 0.5). Effect sizes were estimated based on previously published data evaluating interlimb differences in similar samples with ACLR. To be included, participants were required to be cleared by a physician for return to physical activity and to engage in exercise for at least 30 minutes 3 times per week. Participants were excluded for ACL reinjury (re-tear or revision surgery), bilateral ACLR, any lower extremity injury within 6 months prior to participation, or history of lower extremity surgery other than ACLR. The study was approved by the university’s institutional review board (California State University, Fullerton; Fullerton CA, USA) and all participants provided written informed consent prior to participation.

### Ultrasound Imaging

Imaging was performed by a researcher with more than 10 years experience in musculoskeletal ultrasound imaging who was originally trained by a physician in primary care (sports medicine). All participants were instructed to avoid exercise immediately prior to the investigation to limit compression of articular cartilage. Upon arrival at the lab, participants lay down in a non-weightbearing supine position, prior to ultrasound, with their knees fully extended for 30 minutes to unload femoral cartilage. A Logiq E ultrasound device with a 12-5 MHz linear array transducer was used to image femoral cartilage (frequency: 12 Hz, depth: 4.0 cm, and gain: 50). While supine, participants had their knees positioned in random order, using a random number generator at 140° or 90° of flexion using a handheld goniometer. These positions were chosen because they are prominent within the literature^21,28^ and the difference between positions exceed typical measurement error of handheld goniometry.^29^ Both positions expose a load-bearing portion of distal anterior femoral articular cartilage with limited obstruction from surrounding soft tissue. Maximum knee flexion has been used in previous research as a position to view femoral cartilage during ultrasound image acquisition.^9,30^ However, this position can alter between participants by up to 17°.^9^ To consistently measure the same portion of cartilage, so as to accurately compare positions, a standardized 140° of flexion was chosen as this is attainable for most individuals and still exceeds handheld goniometry error^29^ to ensure we are measuring 2 distinct portions of femoral cartilage. The ultrasound probe was placed in the transverse plane superior to the patella over the medial and lateral condyles of the femur. The intercondylar notch was centered on the screen using a transparent grid that was overlaid on the ultrasound screen. Three images of the knee at both positions were obtained from the ACLR and contralateral limb in a block random order.

### Gait Biomechanics

Bilateral static markers were placed on the anterior superior iliac spine (ASIS), greater trochanter, iliac crest medial and lateral femoral condyles medial and lateral malleoli, calcaneus, and first and fifth metatarsals. Rigid clusters of 4 markers were placed on the sacrum and bilaterally on the thigh, shank, and foot. Three-dimensional gait biomechanics were collected as participants completed 10 overground walking trials over a 10 m runway. All participants wore laboratory-standard neutral cushion footwear (Nike Pegasus 39, Nike Inc., Beaverton OR) and tight-fitting shorts. Marker position and force plate data were acquired using a 9-camera motion capture system (Qualisys, Göteborg, Sweden) and 2 force platforms (AMTI, Germantown MD) sampling at 240 Hz (marker data) and 2400 Hz (force data), respectively. Walking speed was maintained within ±5% of self-selected speed and monitored using infrared timing gates (Tractronix, Belton, MO) placed 1 m apart, surrounding the force plates. Five practice trials were collected and averaged to obtain self-selected walking speed and confirm that participants could strike the force plate without visibly altering their gait. Participants were instructed to continue walking after contacting the force plate to avoid deceleration. Participants were also instructed to look straight ahead and avoid looking down to target the force plates. All test trials were deemed acceptable if (1) the entire foot contacted the force platform, (2) participants did not exhibit visible gait deviations, and (3) walking speed was within ±5% of self-selected pace. Five valid trials were recorded for each participant. Force platforms were positioned, such that data for both limbs could be obtained from a single trial.

### Data Reduction

**Figure 1.**
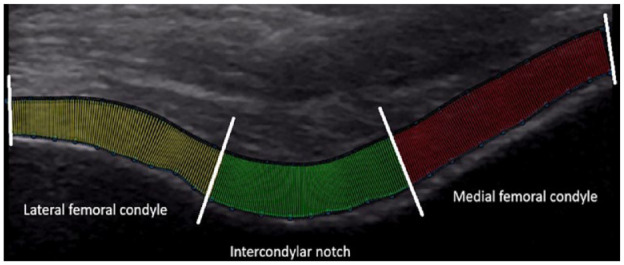
The hyperechoic cartilage-bone interface segmented into medial, central, and lateral portions of the distal femur using the Euclidian distance of 300 evenly spaced data points to measure average cartilage thickness (mm) and to measure echo intensity (0-255). Red indicates the medial compartment, green indicates the central compartment, and yellow indicates the lateral compartment. White lines indicate partitions between portions of cartilage.

Ultrasound images were analyzed using a custom MATLAB program by an investigator who was blinded to limb injury status. The cartilage borders were manually segmented and fit to polynomials, and 300 coordinates were interpolated along each border. Afterward, the Euclidian distance from the hyperechoic cartilage-bone interface to the synovial space-cartilage interface was calculated between pairs of data points along each border to measure thickness, respectively. The average from data points 1-100, 101-200, and 201-300 represented average thickness of the medial, central, and lateral regions of the anterior distal femur, respectively (**Fig. 1**). EI was assessed through tracing the cartilage cross-sectional area from the same portions of cartilage. EI is obtained using grayscale analysis, with EI values being expressed between 0 (black) and 255 (white). Water content within cartilage appears hypo-echoic and thus contributes to a lower EI.

**Figure 2.**
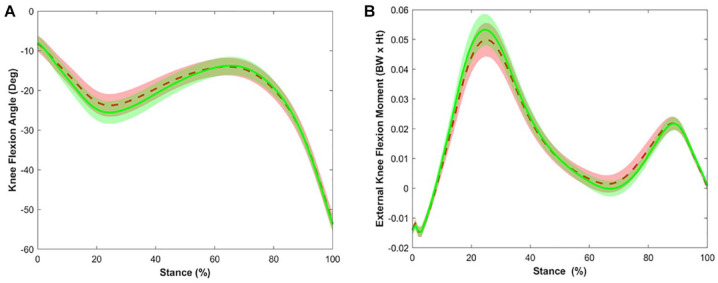
Ensemble average waveform and 95% confidence intervals over the stance phase of gait for the knee flexion angle (**A**) in degrees and external knee flexion moment (**B**) in Nm normalized to a product of body weight and height (BW*Ht): Red dashed line indicates injured limb, and green solid line indicates uninjured limb.

Marker position and force plate data were exported to Visual 3D (C-Motion Inc., Germantown, MD) for model construction and low-pass filtered at 6 Hz, using a fourth order zero-phase lag digital Butterworth filter based on residual analyses. Ankle and knee joint centers were estimated as the midpoints between the medial and lateral malleoli and femoral epicondyles. The hip joint center was estimated using the Davis method.^31^ A joint coordinate system was used to derive knee angles and defined as motion of the shank relative to the thigh.^32^ External knee joint moments were calculated using inverse dynamics and resolved in the tibial coordinate system, and normalized to a product of body weight and height. The stance phase was identified as the interval from heel strike (vertical ground reaction force [GRF] >20 N) to toe off (vertical GRF <20 N). A custom LabVIEW program (National Instruments, Austin, TX) extracted outcomes throughout the stance phase. Outcomes included peak KFM and peak KFA from the first 50% of the stance phase, and ensemble average waveforms were time normalized to 101 data points for visualization purposes (**Fig. 2**). The average of 5 trials from each limb were used for analyses.

### Statistical Analyses

Statistical analyses were completed using SPSS Version 29 (IBM, Armonk, NY). Data were inspected for normality using a Shapiro-Wilk test and assessed for outliers using box plots. Intrarater reliability of ultrasound measures were assessed since images were manually segmented using intraclass correlation (ICC_3,1_), and ICCs were classified as low (< 0.5), moderate (0.5-0.69), and good (> 0.7).^33^ Measurement precision was evaluated using standard error of the measurement and calculated as follows: SEM = SD × √(1 – ICC). Cartilage outcomes were compared between imaging positions and limb using a 2 (*Position*: 90°, 140°) × 2 (*Limb*: ACLR, contralateral) repeated measures analysis of variance (ANOVA; α = .05). *Post hoc* comparisons were conducted using dependent samples *t* tests, using a Bonferroni correction. Although not a study purpose, gait characteristics were compared using a paired sample *t* test to contextualize our findings. Linear regression was used to evaluate the unique associations between gait biomechanics and cartilage outcomes in the ACLR limb based on previously published data finding linear associations between outcomes.^21,22^ Covariates were entered first, followed by biomechanical variables in separate models. Covariates included sex and time since ACLR because time since ACLR influences KOA development^34^ and sex influences gait biomechanics.^35^ Gait speed was not included as a covariate because it expressed collinearity with gait kinetics (variance inflation factor = 2.31-2.42). A significant association was based on the change in explained variance (ΔR^2^ α = .05).

## Results

All data were normally distributed and treated as such (Shapiro-Wilk statistic range: 0.929-0.985, *P* value range: 0.055-0.949), and no outliers were identified. Good to excellent intrarater reliability (ICC range: 0.73-0.92) was found for all ultrasound-derived outcomes (**Table 1**).

**Table 1.** Intrarater Reliability Analyses (Interclass Correlation [ICC] and 95% Confidence Interval, and Standard Error of Measurement [SEM]).

	90°		140°	
	ICC_3,1_	SEM	ICC_3,1_	SEM
Medial thickness (mm)	0.88 (0.81, 0.92)	0.13	0.88 (0.81, 0.92)	0.12
Central thickness (mm)	0.92 (0.87, 0.95)	0.19	0.91 (0.86, 0.94)	0.12
Lateral thickness (mm)	0.73 (0.61, 0.82)	0.18	0.80 (0.70, 0.88)	0.11
Medial echo intensity (0-255)	0.73 (0.62, 0.82)	9.84	0.86 (0.75, 0.93)	6.12
Central echo intensity (0-255)	0.75 (0.64, 0.84)	5.61	0.85 (0.74, 0.92)	4.92
Lateral echo intensity (0-255)	0.76 (0.65, 0.84)	9.70	0.86 (0.79, 0.91)	8.11

Three images were obtained and each was manually segmented by a single investigator. ICC_3,1_ (2-way mixed, single measure) were obtained from 3 measurements (1 per image). The SEM was calculated to estimate measurement precision.

## Interlimb Cartilage Comparison

There were no position × limb interactions for any cartilage outcome (all *P* > 0.05, **Table 2**). There was a main effect of position on medial cartilage thickness (F_1-26_ = 4.776, *P* = 0.038, $\eta_{p}^{2}=.155$ ), central cartilage thickness (F_1-26_ = 40.912, *P* < 0.001, partial $\eta_{p}^{2}=.611$ ), central EI (F_1-26_ = 5.317, *P* = 0.029, $\eta_{p}^{2}=.170$ ) and lateral EI (F_1-26_ = 10.413, *P* = 0.003, $\eta_{p}^{2}=.286$ ).

**Table 2.** Cartilage Thickness Measurements for Cartilage in Both the Anterior Cruciate Ligament Reconstructed (ACLR) and Contralateral Limb.

	Contralateral Limb		ACLR Limb				*P*
	90°	140°	90°	140°	Limb × Position	Limb	Position
Medial thickness (mm)	2.02 ± 0.40	1.85 ± 0.29	2.14 ± 0.42	2.06 ± 0.40	0.37	0.02	0.04
Central thickness (mm)	2.95 ± 0.51	2.11 ± 0.36	2.97 ± 0.80	2.32 ± 0.46	0.17	0.21	<0.01
Lateral thickness (mm)	2.06 ± 0.33	1.91 ± 0.29	2.08 ± 0.46	2.03 ± 0.31	0.26	0.22	0.08

Medial cartilage (mean difference = 0.121 mm [SE: 0.056]) and central cartilage (mean difference = 0.745 mm [SE = 0.116]) were thicker when measured at 90° compared with 140°.

Central (mean difference = −4.205 [SE=1.824]) and lateral EI (mean difference = −8.058 [SE = 2.497]) were lower (darker) at 90° compared with 140° (**Table 3**).

**Table 3.** Echo Intensity Measurements for Cartilage in Both the Anterior Cruciate Ligament Reconstructed (ACLR) and Contralateral Limb.

	Contralateral Limb		ACLR Limb				*P*
	90°	140°	90°	140°	Limb × Position	Limb	Position
Medial EI (0-255)	24.16 ± 17.49	26.76 ± 19.57	30.16 ± 20.85	29.57 ± 20.48	0.58	0.19	0.71
Central EI (0-255)	43.49 ± 12.26	47.78 ± 13.71	46.13 ± 10.69	50.15 ± 11.88	0.91	0.22	0.03
Lateral EI (0-255)	33.04 ± 23.86	40.92 ± 25.80	32.04 ± 19.93	40.27 ± 22.76	0.94	0.79	<0.01

EI = echo intensity.

There was significant main effect of limb for medial cartilage thickness (F_1-26_ = 6.706, *P* = 0.016, $\eta_{p}^{2}=.205$ ) and cartilage was thicker in the ACLR compared with contralateral limb (mean difference = 0.169 mm [SE = 0.016]).

### Interlimb Gait Comparison

There was no significant difference in KFM (t_26_ = −0.80, *P* = 0.432, d = 0.16) between the ACLR (0.05 Nm) and contralateral (0.06 Nm) limb. There was no significant difference in KFA (t_26_ = −0.19, *P* = 0.854, d = 0.04) between ACLR (24.4°) and contralateral (24.2°) limb.

### Association between Gait and Cartilage Outcomes

Covariates explained 19% to 31% of variance in medial (F_2,24_ = 5.317, *P* = 0.012) and central (F_2,24_ = 4.801, *P* = 0.030) cartilage thickness of the ACLR limb when measured at 140°. A larger KFM was associated with thicker medial cartilage in the ACLR limb at 140° (ΔR^2^ = 0.146, *P* = 0.021), and thicker central cartilage in the ACLR limb at 140° (ΔR^2^ = 0.147, *P* = 0.026). Regression analyses are summarized in **Table 4**.

**Table 4.** Regression Summaries Between KFM and Knee Flexion Angle KFA With Cartilage Thickness and EI After Accounting for Sex and Time Since ACLR (Covariates).

Position	Medial			Central			Lateral		
	β	ΔR^2^	*P*	β	ΔR^2^	*P*	β	ΔR^2^	*P*
Thickness measurements									
90°									
KFM	3.611	0.006	0.688	14.013	0.026	0.432	7.125	0.509	0.483
KFA	0.001	0.001	0.968	−0.005	0.002	0.841	0.006	0.006	0.701
140°									
KFM	16.775	0.146	0.021^a^	19.418	0.147	0.026^a^	8.842	0.067	0.117
KFA	−0.008	0.014	0.491	0.009	0.014	0.511	−0.006	0.015	0.470
EI measurements									
90°									
KFM	168.593	0.005	0.719	151.328	0.017	0.484	−77.74	0.001	0.864
KFA	−0.864	0.063	0.215	0.126	0.005	0.701	0.342	0.011	0.616
140°									
KFM	239.714	0.011	0.597	−124.888	0.009	0.590	−294.094	0.014	0.547
KFA	0.486	0.021	0.477	−0.126	0.004	0.720	−0.404	0.012	0.585

KFM and KFA were added independently after accounting for covariates, and the change in coefficient in determination (ΔR)^2^ was used to assess the strength and significance of association.

KFM = knee flexion moment; KFA = knee flexion angle; ACLR = anterior cruciate ligament reconstruction; EI = echo intensity.

a Δ*P* < 0.05.

## Discussion

The purpose was to compare femoral cartilage thickness and EI when measured at 90° and 140° of knee flexion, and between limbs of individuals with unilateral ACLR. Our primary findings indicate that medial and central cartilage is thicker, and central and lateral cartilage EI was lower when measured at 90° compared with 140°. Medial cartilage was thicker in the ACLR compared with contralateral limb, regardless of measurement position. We also found that a larger KFM was associated with thicker medial and central cartilage in the ACLR limb when measured at 140°.

We found that medial and central cartilage was thicker and central and lateral cartilage EI was lower when measured at 90° than at 140° degrees of knee flexion that exceeded the SEM, which partially supported our hypotheses. As knee flexion increases, the patella translates inferiorly with respect to the femur, resting below the intercondylar groove.^36^ Therefore, an inferior shift of the patella provides a view of a more inferior aspect of the distal femur the further the knee is flexed. Femoral cartilage morphology is affected by compressive forces,^37^ which are not dispersed equally across the distal femur during activities such as walking.^38^ Previous research has shown that cartilage T2 relaxation times, a measure of integrity of the collagen matrix and changes in cartilage water content, are significantly different between the anterior and central femur.^39^ EI has been theorized to evaluate cartilage water content.^40^ Increased cartilage water content is related to changes in cartilage composition, which can reduce the capability of cartilage to respond appropriately to increased loading.^41^ As water is hypo-echoic, this study indicates that central and lateral cartilage measured at 90° of knee flexion may have greater water content compared with that at 140°, which may be indicative of a cartilage composition that is less resistant to deformation. However, we interpret these results with caution as the difference in EI between measurement positions did not exceed the SEM. Future studies are needed with larger samples to definitively compare femoral cartilage EI between limbs with and without ACLR.

Femoral cartilage lesions are commonly found in areas of patellofemoral contact that occur between 40° and 80° of knee flexion in individuals with OA.^42^ This area reflects the central and inferior portion of the anterior distal femur and is also the region of thickest cartilage in individuals without OA.^37^ This information may inform optimal positions for assessing femoral cartilage morphology and explain why cartilage was thinner when imaging at 140° compared with 90°. If the patella translates inferiorly over the femur during maximum knee flexion, the portions of thickest cartilage may be outside of the field of view. Rather, a more central portion of the femur may be visible, which has thinner cartilage.^43^ Our data suggest that measurement position influences femoral cartilage features assessed with ultrasound and should be considered when implementing ultrasound imaging in future study designs or clinical practice.

Thicker cartilage was found on the medial distal femur in the ACLR, compared with contralateral limb in both measurement positions that exceeded the SEM, which did not agree with our hypothesis. Our hypothesis was based on previous data that cartilage loss is a hallmark feature of knee OA^44^ and occurs more frequently within the reconstructed limb of individuals with ACLR compared with contralateral limb.^3^ However, there is evidence that individuals with early stage knee OA may experience acute cartilage thickening.^45^ A study using ultrasound found thicker medial and lateral femoral cartilage in the ACLR limb compared with the contralateral limb in participants that were 37 months from reconstruction.^46^ Cartilage swelling may occur before cartilage loss due to damage to the collagen network, which reduces its elastic restraint and causes glycosaminoglycans within cartilage to increase hydration.^47^ Water content and the extracellular matrix provides a portion of cartilage compressive stiffness, and alterations in either may contribute to variations in chondrocyte volume and cartilage structure.^48^ Therefore, cartilage thickness should be examined in future prospective studies to determine the timeline of cartilage breakdown after ACLR.

A larger KFM was associated with thicker cartilage in both the medial and central portions of the distal femur when measured at 140° of knee flexion, which partially agreed with our hypothesis. Individuals with ACLR commonly walk with a smaller KFA and KFM due to quadriceps dysfunction, and a higher KFM contributes to greater patellofemoral and medial tibiofemoral joint compressive force.^48,49^ It is theorized that positioning the knee in maximum flexion exposes a central portion of cartilage that is influenced by patellofemoral and tibiofemoral loading, which may explain why a larger KFM was associated with thicker medial cartilage at 140° but not at 90° of knee flexion. Mechanical loading from the KFM is necessary to maintain cartilage health due to the tissue’s mechanosensitive nature, and joints that experience higher levels of contact pressure have thicker cartilage layers.^50^ No differences in peak KFM were found between the ACLR and contralateral limb in our sample, which may explain why thicker cartilage was found within the ACLR limb. No associations were found between lower KFM and thinner anterior femoral cartilage when viewed at 90°. It is possible that a greater KFM may affect this portion of cartilage differently, possibly due to overloading within the patellofemoral joint, resulting in cartilage thinning about the anterior femur. Further longitudinal research outlining timelines of cartilage thickness change within this portion of the femur, and their associations with gait biomechanics, would be required to test this hypothesis.

The distal anterior femur articulates with the patella and tibia throughout the knee’s full range of motion.^51^ Previous literature suggests that the purpose of using maximum knee flexion during ultrasound imaging is to provide an obstructed view of the weightbearing surface of the femoral condyles.^9^ Conversely, Schmitz et al (2017) found that a larger internal knee extension moment was associated with greater medial femoral cartilage thickness when measuring femoral cartilage at 90° of knee flexion using ultrasound. Differences in findings may be attributable to probe placement. A suprapatellar transverse probe placement at 140º and 90º was used in this study to view 3 regions (medial, central, and lateral) at 2 distinct portions of the anterior femoral cartilage. Schmitz *et al.* placed the probe transversely over the medial femoral condyle rather than superior to the patella, which may provide a view of the central weightbearing portion of the medial femoral condyle. When imaging cartilage superior to the patella, it is possible that 90° of knee flexion does not expose a portion of the distal femur that is influenced by peak KFM during gait. However, tasks that utilize different joint kinematics (e.g., running, jumping) may provide a mechanical stimulus that favors other regions of the knee joint.^52^ Therefore, further studies should evaluate whether other tasks (e.g., running) or other gait kinetics (e.g., tibiofemoral compressive forces, knee adduction moment, and second KFM peak) are associated with cartilage morphology at different positions or probe placements during ultrasound image acquisition.

There are limitations to consider when interpreting the findings of this research. First, the cross-sectional study design impairs the ability to ascertain whether gait biomechanics or ACLR determine changes in cartilage characteristics. Second, the cartilage measurements for thickness and EI were taken from cross sections of the lateral, central, and medial regions of the ultrasound image obtained and are limited by probe width. However, it should be noted that the extreme convex surface of the femur outside of the probe width does not contact the tibia or patella and is likely of little interest when evaluating cartilage thickness in the tibiofemoral or patellofemoral joint. Third, the sample was heterogeneous in terms of graft type and meniscal injury, which may influence cartilage and gait characteristics. Individuals with patellar tendon grafts have smaller KFMs at 6 to 12 months post-ACL surgery than controls and individuals with hamstring grafts.^53^ However, a recent meta-analysis found that, when comparing ACLR and control participants, there was no effect of graft type on gait outcomes (KFM and knee adduction moment) ≥1 year after ACLR.^54^ Meniscal injury has been associated with increased onset of knee OA within an ACLR population^52^ and has also been seen to affect gait biomechanics post-surgery.^55^ However, the self-report nature of the study and variability in the type of meniscus injury and surgical procedure (meniscectomy or repair) make it difficult to confidently incorporate them in analyses. External joint moments were used as a surrogate of knee joint loading and may not comprehensively reflect internal knee loading. For instance, individuals with ACLR may have elevated hamstrings co-contraction^56^ during gait, which may contribute to greater knee loading. Further research should consider models that more accurately quantify internal joint kinetics to further our understanding of how aberrant gait mechanics influence cartilage features after ACLR. Finally, this study only compared the ACLR limb with the contralateral limb within individuals and did not consider a control group without injury history. Future studies should consider a control group that can elucidate how ACLR influences the magnitude of interlimb differences in cartilage outcomes.

## Conclusion

Altering knee position during ultrasound image acquisition exposes a different portion of the distal femur that varies in cartilage thickness and EI. As such, researchers and clinicians should be cognizant of measurement position when interpreting cartilage outcomes obtained using ultrasound imaging. Individuals with ACLR had thicker cartilage in their ACLR limb compared with contralateral limb, which may indicate cartilage swelling. Longitudinal studies are needed to determine timelines of cartilage degeneration after ACLR. The KFM is associated with cartilage thickness at specific portions of the anterior distal femur. Therefore, knee loading during gait may not be consistently applied across the joint surface and contribute to regional differences in cartilage morphology and composition. Studies are needed that comprehensively evaluate the unique longitudinal relationships between knee biomechanics and cartilage throughout the entire knee.
